# Single-molecule real-time sequencing of the full-length transcriptome of loquat under low-temperature stress

**DOI:** 10.1371/journal.pone.0238942

**Published:** 2020-09-11

**Authors:** Cuiping Pan, Yongqing Wang, Lian Tao, Hui Zhang, Qunxian Deng, Zhiwu Yang, Zhuoheng Chi, Yunmiao Yang

**Affiliations:** 1 College of Horticulture, Sichuan Agricultural University, Wenjiang, Sichuan, China; 2 Horticulture Institute, Sichuan Academy of Agricultural Sciences, Chengdu, Sichuan, China; Huazhong University of Science and Technology, CHINA

## Abstract

In this study, third-generation full-length (FL) transcriptome sequencing was performed of loquat using single-molecule real-time(SMRT) sequencing from the pooled cDNA of embryos of young loquat fruit under different low temperatures (three biological replicates for treatments of 1°C, -1°C, and -3°C, for 12 h or 24 h) and the control group(three biological replicates for treatments of room temperature), Illumina sequencing was used to correct FL transcriptome sequences. A total of 3 PacBio Iso-Seq libraries (1–2 kb, 2–3 kb and 3–6 kb) and 21 Illumina transcriptome libraries were constructed, a total of 13.41 Gb of clean reads were generated, which included 215,636 reads of insert (ROIs) and 121,654 FL, non-chimaric (FLNC) reads. Transcript clustering analysis of the FLNC reads revealed 76,586 consensus isoforms, and a total of 12,520 high-quality transcript sequences corrected with non-FL sequences were used for subsequent analysis. After the redundant reads were removed, 38,435 transcripts were obtained. A total of 27,905 coding DNA sequences (CDSs) were identified, and 407 long non-coding RNAs (lncRNAs) were ultimately predicted. Additionally, 24,832 simple sequence repeats (SSRs) were identified, and a total of 1,295 alternative splicing (AS) events were predicted. Furthermore, 37,993 transcripts were annotated in eight functional databases. This is the first study to perform SMRT sequencing of the FL transcriptome of loquat. The obtained transcriptomic data are conducive for further exploration of the mechanism of loquat freezing injury and thus serve as an important theoretical basis for generating new loquat material and for identifying new ways to improve loquat cold resistance.

## Introduction

Loquat (*Eriobotrya japonica* Lindl) originated in China and has been cultivated for 2100 years. Owing to its economic and ecological attributes, loquat is an important perennial fruit crop species and is cultivated largely between the N 35° and S 35° latitudes worldwide [1–2]. Loquat blossoms in late autumn or early winter, and young fruits are vulnerable to freezing injury [3–5]. In 2016, 90% of the loquat planting area in China experienced freezing, with almost no material harvested. Freezing injury has severely jeopardized the economic benefits of farmers and has become a major restricting factor for sustainable development in many production areas worldwide. Current research on loquat has mainly focused on cell genetics [6, 7], physiology and biochemistry [8, 9], molecular markers [10], molecular clones [11, 12], etc. Several transcriptome studies in loquat focused on flower bud differentiation [13], fruit development and ripening [14, 15], and postharvest storage [16], research on transcriptome in cold stress of loquat is limited [17], little is known about its cold tolerance mechanisms. Previous studies have been performed using second generation sequencing technology, and many unigenes have been obtained, however, transcriptomic sequences using second generation sequencing technology may be misassembled without a high-quality genome sequence or full-length (FL) transcriptomic sequences available as a reference [18]. To date, FL transcriptomic data are scarce. In addition, Loquat is a non-model plant species with high heterozygosity, and a loquat reference genome is still lacking, which has limited molecular biological research of this species.

In recent years, third-generation sequencing technology has been successfully applied to functional genomics research of sweet potato [19], Populus [20], sorghum [21], corn [22], and cotton [23], among others. Compared with second-generation sequencing technology,third-generation sequencing technology not only has advantages that include handling a large volume of data and the ability to read long sequences and FL gene transcripts, but it is also greatly more accurate in terms of gene functional annotation without sequence splicing and assembly [24].

In the present study, The FL transcriptome of embryos of young loquat fruit under low-temperature stress was obtained by single-molecule real-time (SMRT) sequencing. This work will facilitate future research on identifying functional genes and analysing molecular mechanisms related to the cold stress response of loquat.

## Materials and methods

### Plant materials and treatments

Two-year-old grafted Ninghaibai loquat plants that were growing in pots and that had already produced fruit (with a diameter of approximately 1.5 cm) were used as the experimental materials, and the growth status of the plants was as uniform as possible. The plants were subjected to three different temperatures, 1°C, -1°C, and -3°C, for 12 h or 24 h separately after being subjected to a gradient of cooling at a rate of 4°C/h. The treatments were applied in a low-temperature plant incubator with 60% relative humidity, a 3000 lx light intensity, and a 12-h/12-h light/dark cycle. The plants were then removed and incubated at room temperature for 6 h to recover, after which the embryos of young loquat fruit were collected, immediately frozen in liquid nitrogen and stored at -80°C.

Plants that had been growing at room temperature were used as controls. Each treatment involved three biological replications. A total of 21 samples of embryos of young loquat fruit (three biological replicates for treatments of 1°C, -1°C, and -3°C, for 12 h or 24 h, including the control group) were collected for the following experiments.

### RNA extraction and quantification

Total RNA was extracted with the RNAprep Pure Plant Kit (TIANGEN, Cat. No. DP441) following the manufacturer’s protocol. The samples were quantified as follows. The purity and concentration of RNA were first measured using a NanoDrop ND-1000 spectrophotometer (NanoDrop Technologies, Rockland, DE, USA) according to their OD260/280 value, after which the RNA integrity was assessed using an RNA Nano 6000 Assay Kit in conjunction with an Agilent Bioanalyzer 2100 system (Agilent Technologies, CA, USA). The RNA degradation and contamination were measured on 1% agarose gels. Only total RNAs with a RIN score ≥8.0 were used to construct cDNA libraries for SMRT or Illumina sequencing.

### PacBio Iso-Seq library preparation and sequencing

After the RNA quality was verified, libraries were constructed. mRNA was purifed from 3μg of mixed total RNA of 21 samples of embryos of young loquat fruit for SMRT library preparation and sequencing. The instruments used include a SMARTer^™^ PCR cDNA Synthesis Kit (Clontech, CA, USA)and BluePippin^®^ Size Selection System (Sage Science, Beverly, MA, USA). The SMARTer^™^ PCR cDNA Synthesis Kit (Clontech, CA, USA) was used for synthesizing FL cDNA, the generated cDNAs were then reamplified via PCR. The remaining overhangs were converted to blunt ends by exonuclease/polymerase activities. After adenylation of the 3′ ends of the DNA fragments, NEBNext Adaptors with a hairpin loop structure were ligated in preparation for hybridization. The BluePippin^®^ Size Selection System was used for size selection(1–2 kb, 2–3 kb and 3–6 kb) to bulid 3 libraries.

The quality of the libraries was assessed using an Agilent Bioanalyzer 2100 system, and SMRT sequencing was performed using a Pacific Biosciences real-time sequencer in conjunction with C2 sequencing reagent.

### Illumina transcriptome library preparation and sequencing

21 second-generation-sequencing cDNA libraries of embryos of young loquat fruit (three biological replicates for treatments of 1°C, -1°C, and -3°C, for 12 h or 24 h, including the control group) were constructed respectively using a NEBNext^®^ Ultra^™^ RNA Library Prep Kit for Illumina^®^ (NEB, Beverly, MA, USA) according to the manufacturer’s protocol. Briefly, mRNA was purified from 5μg of total RNA using poly-T oligo-attached magnetic beads. Fragmentation was carried out using divalent cations under high temperature in NEBNext First Strand Synthesis Reaction Buffer (5X). First-strand cDNA was synthesized using random hexamer primers and M-MuLV Reverse Transcriptase (RNase H-). Second-strand cDNA synthesis was subsequently performed using DNA polymerase I and RNase H. The remaining overhangs were converted to blunt ends via exonuclease/polymerase activities. After poly-adenylation of the 3’ ends of the DNA fragments, NEBNext adaptors with hairpin loop structures were ligated in preparation for hybridization. An AMPure XP system (Beckman Coulter, Beverly, USA) was used to select cDNA fragments that were 200–250 bp in length. Afterward, 3 μl of USER Enzyme (NEB, USA) together with size-selected, adaptor-ligated cDNA was incubated at 37°C for 15 min and again at 95°C for 5 min. PCR was then performed with Phusion High-Fidelity DNA Polymerase, universal PCR primers, and Index (X) Primer. The PCR products were ultimately purified (AMPure XP system), and the library quality was assessed using the Agilent 2100 system. The qualified libraries were pair-end sequenced on an Illumina HiSeq 2500 (Illumina, San Diego, CA, USA) system.

### Error correction and quality control of SMRT reads

Raw data (raw reads) in fastq format were first processed using in-house Perl scripts. Raw SMRT sequencing reads were processed by removing polymerase reads that were <50 bp and had a accuracy <0.8, resulting in subreads. The joined subreads were disconnected, and joint sequences that were <50 bp were removed, resulting in clean data. The obtained clean reads were processed into error-corrected reads of inserts (ROIs) with parameters including full passes ≥0 and a sequence accuracy ≥0.8. Then, full-length, non-chimeric (FLNC) transcripts were determined by searching for poly-A tail signals and the 5’ and 3’ cDNA primers within the ROIs. Iterative clustering for error correction (ICE) [25] was used to obtain consensus isoforms, and FL consensus sequences from ICE were polished using Quiver. High-quality FL transcripts were classified as those with a post-correction accuracy criterion surpassing 99%. Any redundancy in high-quality, FL transcripts was removed by CD-HIT [26], and the integrity of the transcriptome was evaluated without redundancy by BUSCO [27].

### Alternative splicing (AS) detection

We subjected Iso-Seq^™^ data directly to an all-vs-all BLAST analysis [28], with high identity settings. The BLAST alignments that met all the criteria were considered products of candidate AS events [29]. There should be two high-scoring segment pairs (HSPs) in the alignment: two HSPs had the same forward/reverse direction, and within the same alignment, one sequence should be continuous, or with a small "overlap" size (smaller than 5 bp); the other sequence should be distinct to show an "AS gap", and the continuous sequence should align to the distinct sequence almost completely. The AS gap should be larger than 100 bp and at least 100 bp away from the 3'/5' end.

### Simple sequence repeat (SSR) detection

Transcripts >500 bp were selected for SSR analysis using the MIcroSAtellite identification tool (MISA; http://pgrc.ipk-gatersleben.de/misa/http://pgrc.ipk-gatersleben.de/misa/). MISA was used to identify seven SSR types, namely, mononucleotide, dinucleotide, trinucleotide, tetranucleotide, pentanucleotide, hexanucleotide, and compound SSRs, by analysing transcript sequences.

### Prediction of coding DNA sequences (CDSs)

The CDSs and corresponding amino acid sequences within the transcript sequences were predicted using TransDecoder (https://github.com/TransDecoder/TransDecoder/releases). TransDecoder was used to identify candidate protein-coding regions based on the open reading frame (ORF) length, log-likelihood score, nucleotide composition, and (optional) Pfam domain content [30].

### Long non-coding RNA (lncRNA) prediction

Putative protein-coding RNAs were filtered and removed using the following minimum length and exon number thresholds. Transcripts that were longer than 200 nt and that had more than two exons were selected as lncRNA candidates and further screened using the Coding Potential Calculator (CPC) [31]/Coding-Non-Coding Index (CNCI/Coding Potential Assessment Tool (CPAT) [32]/Pfam database, which has the power to distinguish protein-coding genes from non-coding genes.

### Functional annotation of transcripts and analysis of transcription factors (TFs)

The non-redundant transcript sequences obtained were mapped to eight different databases to obtain annotation information associated with the transcripts. These databases included the non-redundant (NR) [33], Swiss-Prot [34], Gene Ontology (GO; http://www.geneontology.org) [35], Clusters of Orthologous Groups of proteins (COG; http://www.ncbi.nlm.nih.gov/COG) [36], euKaryotic Orthologous Groups (KOG) [37], Pfam (http://pfam.janelia.org/) [38], evolutionary genealogy of genes: Non-supervised Orthologous Groups (eggNOG; http://eggnog.embl.de), and Kyoto Encyclopaedia of Genes and Genomes (KEGG, http://www.genome.ad.jp/kegg/) databases [39].

Finally, TFs were predicted using iTAK [40] predictive software.

## Results

### SMRT- and Illumina-based RNA sequencing and error correction

A total of 13.41 Gb of clean data were generated via Pacific Biosciences SMRT sequencing technology. Based on the conditions of full passes ≥0 and a quality >0.8, a total of 215,636 reads of inserts (ROIs)were obtained (Table 1), and 121,654 full-length non-chimeric (FLNC) sequences were identified (Table 2). In total, 76,586 consensus isoforms were obtained by iterative clustering for error correction(ICE) (Table 3). After error correction with second-generation sequencing short reads was performed, a total of 38,435 non-redundant transcripts with an average length of 2607bp were obtained, including 12,520 high-quality transcripts. All the raw data were deposited in the NCBI Sequence Read Archive (SRA) under accession number PRJNA623262 and are available at https://www.ncbi.nlm.nih.gov/bioproject/PRJNA623262.

**Table 1:** ROI statistics.

Samples	cDNA size	Reads of insert	Total read bases of insert	Mean read length of insert	Mean read quality of insert	Mean number of passes
F01	1–2 kb	60768	112445632	1850	0.96	3
F01	2–3 kb	82481	214455943	2600	0.95	7
F01	3–6 kb	72387	244539490	3378	0.94	5
F01	All	215636	571441065	2609.33	0.95	5

**Table 2:** FL sequence statistics.

Samples	cDNA size	Reads of insert	Number of 5’ reads	Number of 3’ reads	Number of poly-A reads	Number of filtered short reads	Number of non-full-length reads	Number of full-length reads	Number of full-length non-chimeric reads	Number of full-length non-chimeric bases	Average length of full-length non-chimeric reads	Full-length percentage (FL%)	Artificial concatemers (%)
F01	1–2 kb	60768	29868	34965	35769	5344	30620	24804	24719	35410268	1432	40.82	0.34
F01	2–3 kb	82481	67290	69380	68092	939	22155	59387	59167	151411494	2559	72.00	0.37
F01	3–6 kb	72387	48656	51394	49502	810	33665	37912	37768	130361660	3451	52.37	0.38
F01	All	215636	145814	155739	153363	7093	86440	122103	121654	317183422	2607	56.62	0.36

**Table 3:** ICE clustering results statistics.

Samples	Number of consensus isoforms	Average read length of consensus isoforms	Number of polished high-quality isoforms	Number of polished low-quality isoforms	Percent of polished high-quality isoforms (%)
F01	76586	2655	12520	63997	16.35

### Predictions of CDSs, lncRNAs, and SSRs

**Fig 1:**
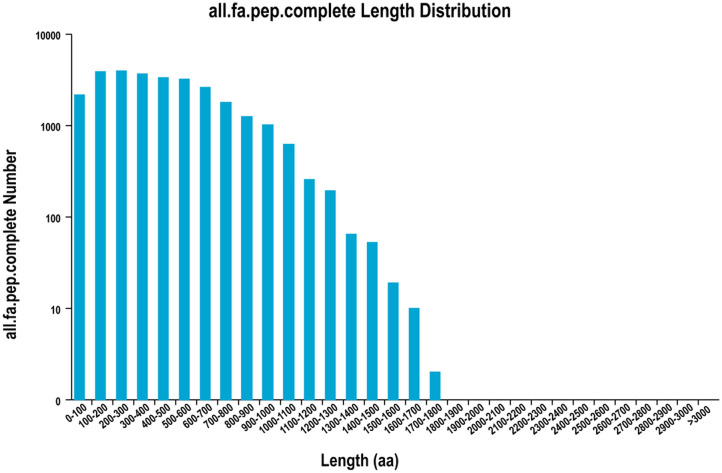
Distribution of the lengths of CDSs within complete ORFs. The x-axis represents the coding sequence length; the y-axis represents the number of predicted ORFs.

**Fig 2:**
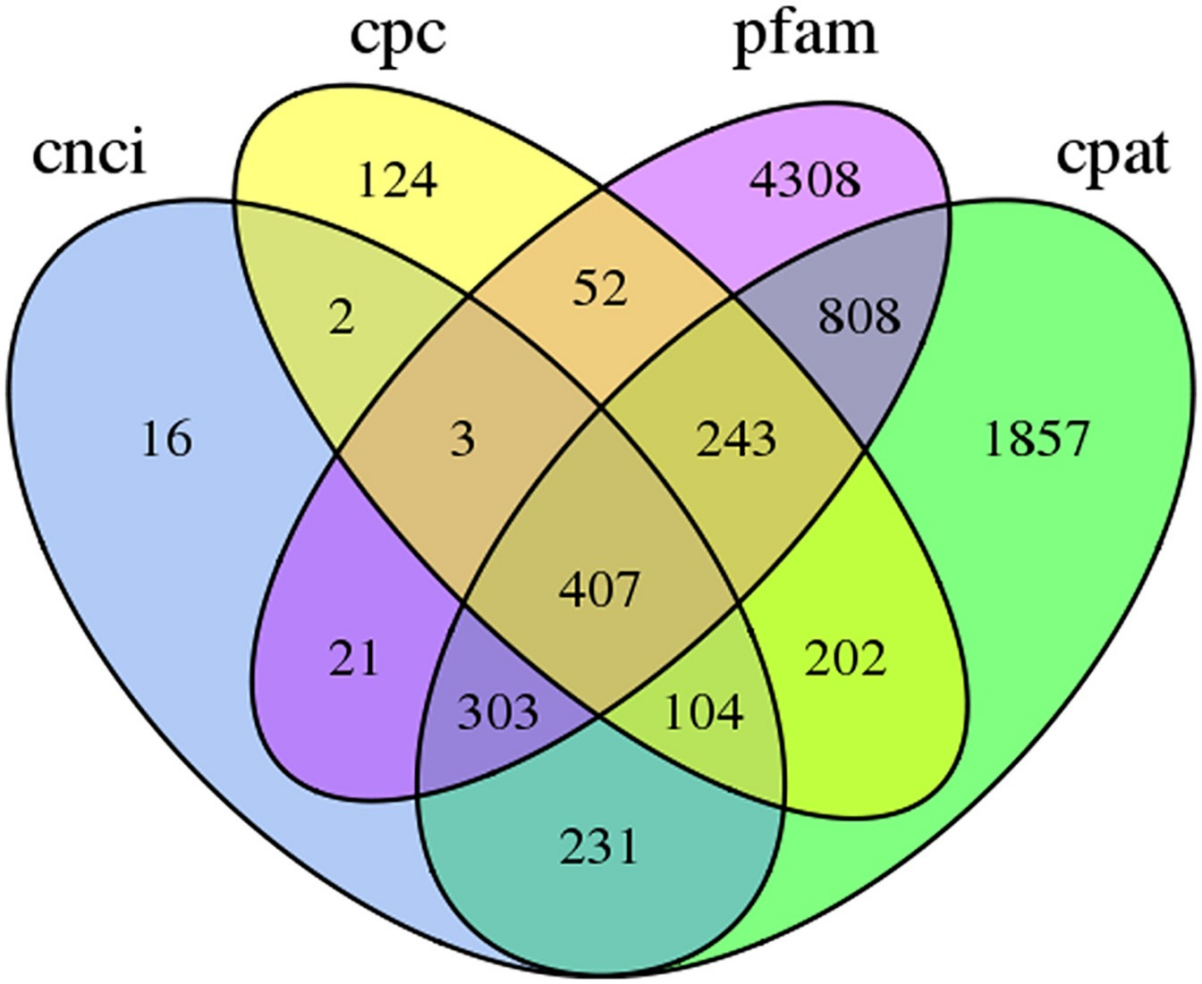
Venn diagram of the number of lncRNAs predicted by CPC, CNCI, CPAT, and Pfam protein structure domain analyses.

A total of 1,295 alternative splicing (AS) sequences were obtained. There were 37,230 ORFs that included 27,905 CDSs identified by TransDecoder, the distribution of the coding sequence lengths of complete ORFs is shown in Fig 1. Four computational approaches (CPC analysis, CNCI analysis, Pfam protein domain analysis, CPAT analysis) were used to screen the transcripts that encode coding proteins (Fig 2), and 407 lncRNAs were predicted. Transcripts that were >500 bp were selected for SSR analysis using MISA. In total, 24,832 SSRs were identified, including 5,317 sequences containing more than 1 SSR and 3,536 SSRs present in compound formation. Moreover, SSRs consisting of one to six (mono-, di-, tri-, tetra-, penta-, and hexa-nucleotides) tandem repeats were identified, Mono-nucleotid repeats (12,230) were the most abundant, followed by di-nucleotid repeats (8857), tri-nucleotid repeats (3327), tetra-nucleotide repeats (254), hexa-nucleotide repeats (95) and penta-nucleotide repeats (69) (Table 4).

**Table 4:** Statistical analysis of the SSR results.

Item	Total number of sequences examined	Total size of examined sequences (bp)	Total number of identified SSRs	Number of SSR containing sequences	Number of sequences containing more than 1 SSR	Number of SSRs present in compound formation	Mono- nucleotides	Di- nucleotides	Tri- nucleotides	Tetra- nucleotides	Penta- nucleotides	Hexa- nucleotides
Numbers	38412	99784911	24832	16390	5317	3536	12230	8857	3327	254	69	95

### Transcript functional annotation and sorting of transcription factors

In total, 37,993 transcripts were annotated in eight databases (Table 5). Among these transcripts, 37,908 were annotated in the NCBI NR database, 16,261 were annotated in the COG database, 22,732 in the GO database, 16,507 in the KEGG database, 24,787 in the KOG database, 31,494 in the Pfam database, 28,599 in the Swiss-Prot database, and 37,074 in the eggNOG database.

**Table 5:** Numbers of annotated transcripts in the publicly available databases.

Annotated databases	Transcript number
Clusters of Orthologous Groups(COG)	16261
Gene Ontology(GO)	22732
Kyoto Encyclopaedia of Genes and Genomes(KEGG)	16507
Eukaryotic Ortholog Groups(KOG)	24787
protein family(Pfam)	31494
The Swiss-Prot Protein Knowledgebase(Swiss-Prot)	28599
Evolutionary Genealogy of Genes: Non-supervised Orthologous Groups(eggNOG)	37074
NCBI Non-Redundant Protein Database(NR)	37908
All annotated	37993
All analysed	38435

**Fig 3:**
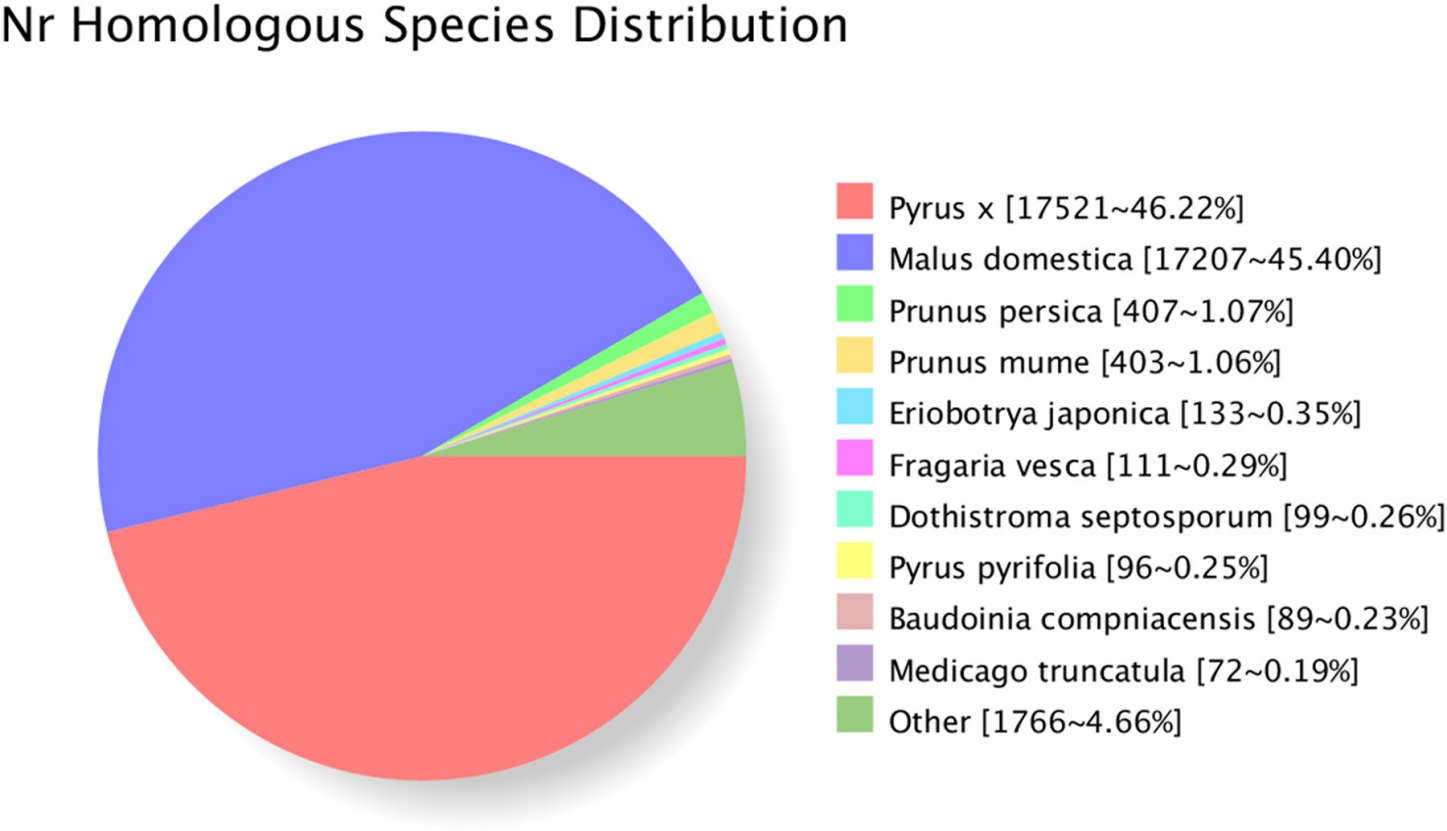
NR annotation of loquat transcripts.

NR contains protein data from the Swiss-Prot, Protein Information Resource, Protein Research Foundation, Protein Data Bank, GenBank, and RefSeq databases;it is a non-redundant protein database housed within the NCBI. The non-redundant transcripts were compared to those in the NR database,the results showed that 46.22% of sequences were aligned to Pyrus x, followed by *Malus domestica*(45.40%), only 0.35% of sequences were aligned to loquat itself (Fig 3).

**Fig 4:**
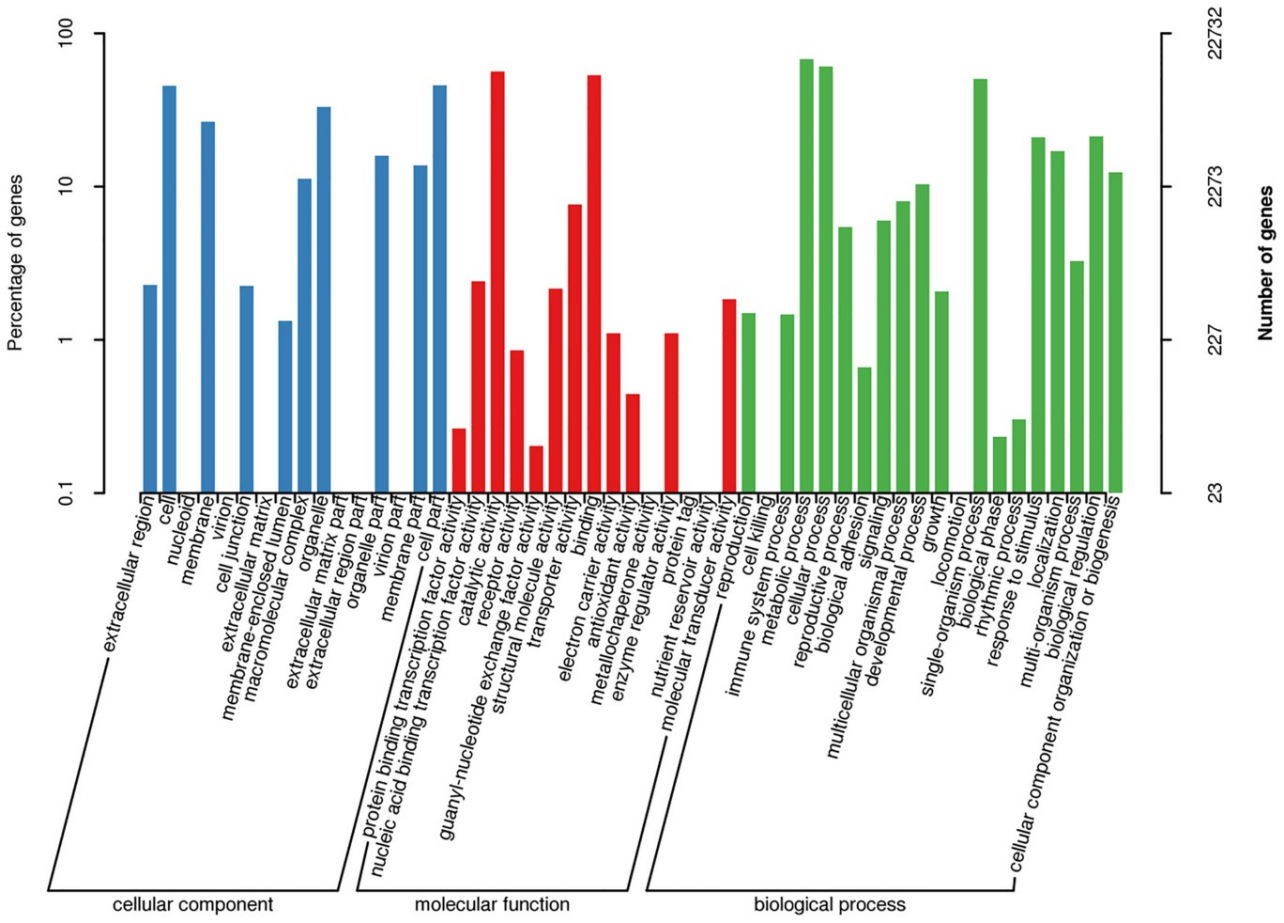
GO functional annotation of loquat transcripts. The x-axis represents GO categories, the y-axis (right) represents the number of transcripts, and the y-axis (left) represents the percentage of transcripts.

GOanalysis indicated that 22,732 transcripts enriched in the pathways related to biological processes, cellular components, and molecular functions. A large number of transcripts in ‘‘cellular components” were mainly involved in cell part, cell, organelle, membrance, membrane part, and macromolecular complex. The category ‘‘molecular functions” mainly consisted of transcripts involved in catalytic activity, binding and transporter activity. The category ‘‘biological process” mainly consisted of transcripts involved in metabolic process, cellular process, single-organism process,biological regulation, localization, responses to stimulus, and cellular component organization or biogenesis (Fig 4).

**Fig 5:**
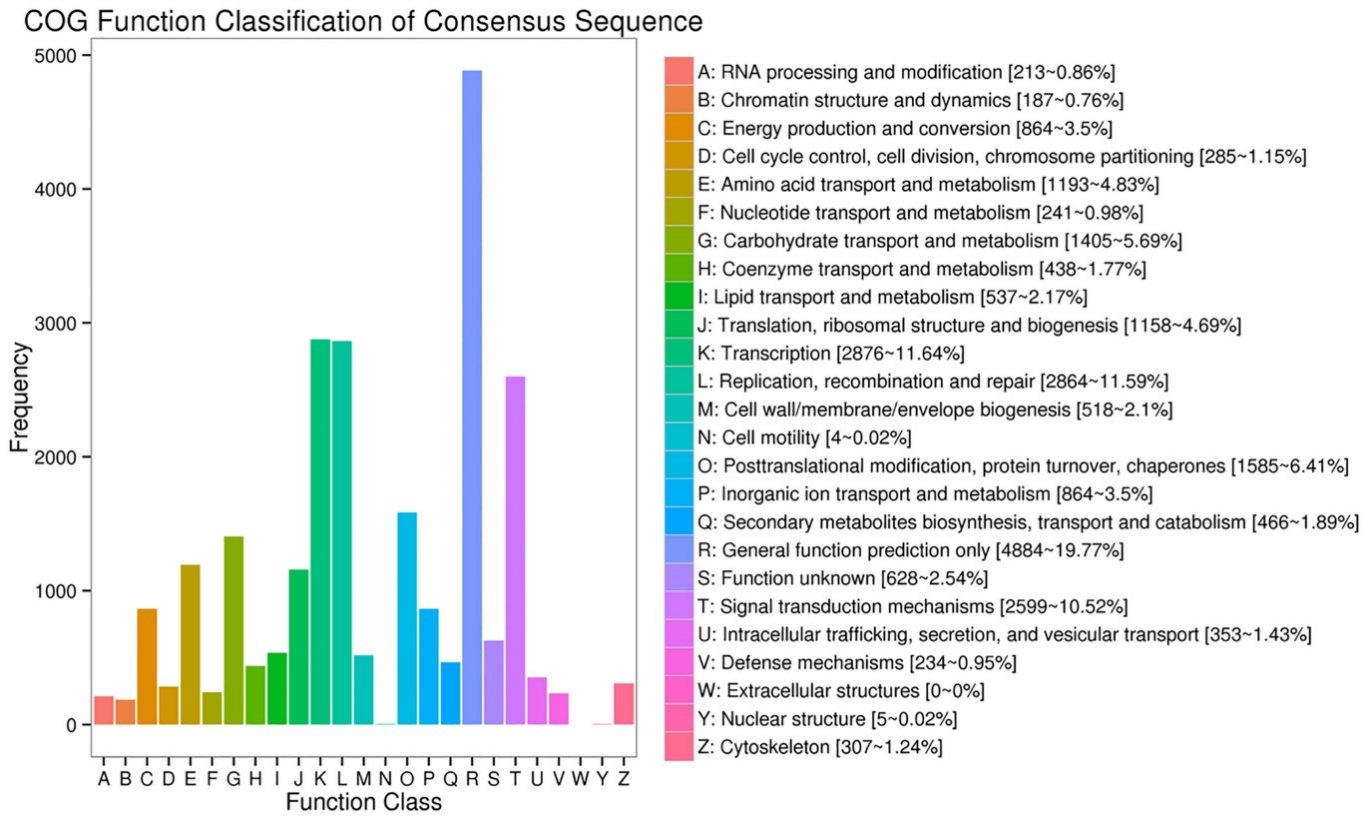
COG annotations of loquat transcripts. The x-axis represents the COG categories, and the y-axis represents the number of transcripts.

In the COG database, we found that the R function (general function prediction only) had the largest number, followed by the K function (transcription), L function (replication, recombination, and repair), and T function (signal transduction mechanisms) (Fig 5).

**Fig 6:**
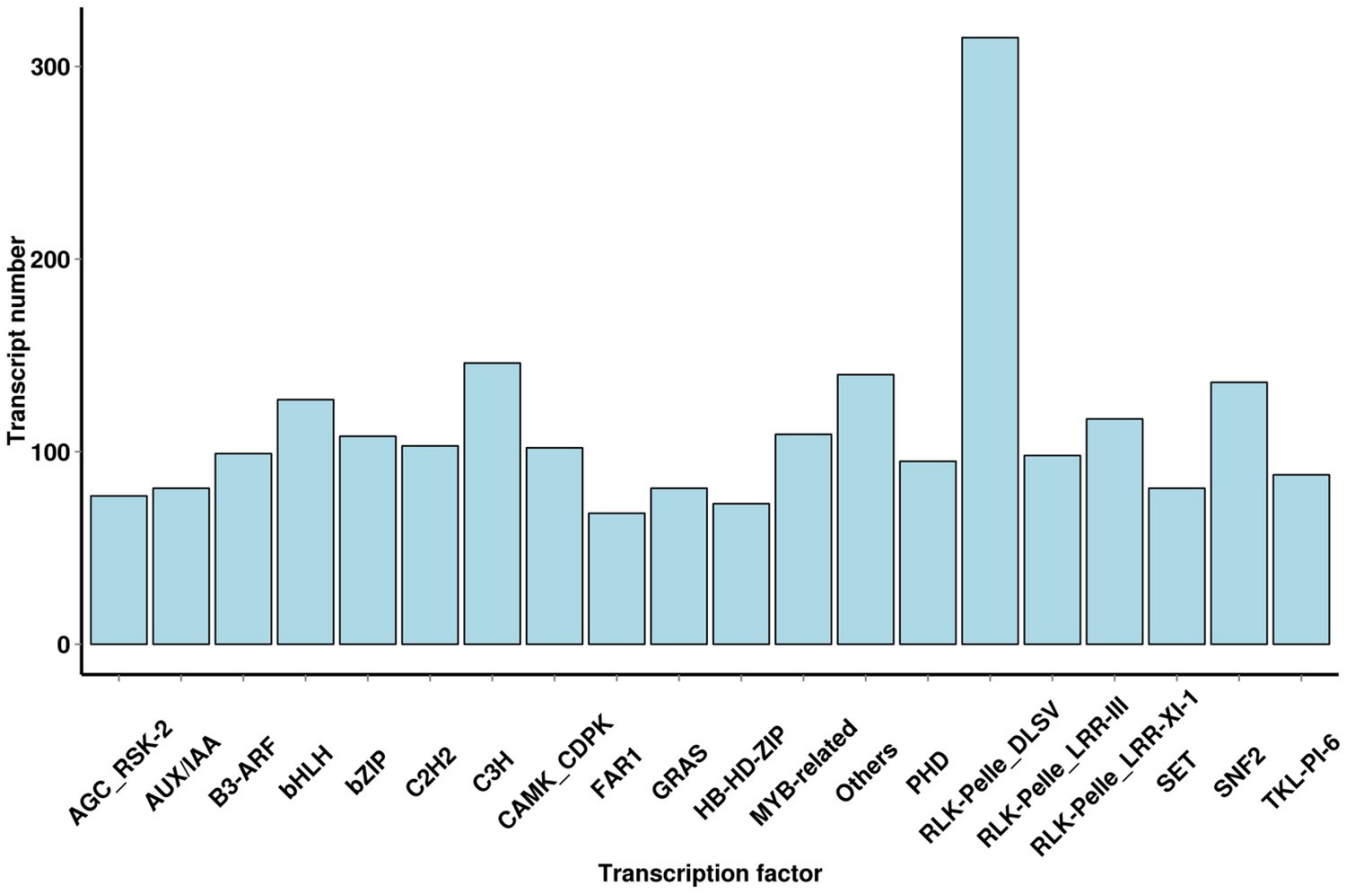
Transcript family of the distribution of TFs. The x-axis represents the type of TF, and the y-axis represents the number of transcripts.

Transcription factors (TFs) play a very important role in the biological processes of plants, A total of 5,322 TFs were predicted by iTAK software, and the numbers of TFs enriched were as follows: RLK-pelle_DLSV (315), C3H (146), SNF2 (136), bHLH (127), and RLK-pelle_LRR-XI-1 (117) (Fig 6).

## Discussion

The loquat genome has yet to be sequenced, research on the physiology and genetics mechanisms of this species has been restricted. Second generation sequencing technology is incapable of assembling full-length transcripts because of the shortness of sequencing reads. AS sites cannot be accurately detected, and the prediction accuracy is lower than 50% [41]. Moreover, fusion genes and gene families cannot be accurately detected. Thus, we can improve the accuracy of transcriptomic data and the prediction accuracy of AS by combining third-generation FL transcriptomic data with second-generation transcriptomic data. Third-generation combined with second-generation sequencing has been widely used to analyze rare transcripts, mining functional genes, analysing different genes in different tissues and at different developmental stages, and analysing the regulatory activity of TFs [42, 43]. To study plants for which a reference genome is not available, the most direct and effective use of ‘omics’ involves transcriptome and digital gene expression profile analysis [44], but obtaining high-quality reference genomes of genetically complex organisms remains costly and is technically challenging [45, 46]. In this study, a total of 13.41 Gb of raw data were obtained by SMRT sequencing, and after clustering analysis, non-FL sequence correction and the removal of redundant sequences, 38,435 transcripts with an average length of 2607 bp were obtained, which is far superior to previous studies of the loquat transcriptome using only the second-generation sequencing technique. For example,Song [47] obtained48,838 transcripts with an average length of 790 bp, and Xu [48] obtained 87,379 transcripts with an average length of 710 bp. Thus, Our findings indicated that SMRT sequencing is an effective route for obtaining reliable full-length transcript sequence information in plants.

LncRNAs are a class of non-coding RNA with a length longer than 200 nucleotides. Currently, many studies have been conducted to examine lncRNAs in animals [49–51], while research on lncRNAs in plants mainly focuses on a few model plants such as Arabidopsis thaliana [52], rice [53], and tomato [54]. In recent years, with the development of high-throughput sequencing technology, an increasing number of studies have focused on lncRNAs in plants, which have been found to play a regulatory role in plant flowering [55], reproductive development [56], photomorphogenesis [57], response to biotic and abiotic stresses [58], and in other biological processes [59]. In the present study, 407 lncRNAs were predicted from the non-redundant transcripts. These newly identifed lncRNAs will be helpful for loquat research in several aspects, and the function of lncRNAs in response to low temperature stress of loquat requires further study.

Full-length sequence transcripts are crucial for genome annotation and gene function research [60]. However, most methods for obtaining full-length transcripts are expensive, time-consuming and inefficient [61]. To date, no full-length sequence transcripts in loquat have been reported. In this study, 38,435 transcripts were obtained using the PacBio SMRT sequencing platform. Based on these transcripts, 37,230 ORFs were predicted, of which 27,905 had a complete CDS, and 37,993 transcripts were annotated into 8 databases including NR, eggNOG, Swiss-Prot, GO, COG, KOG, Pfam and KEGG. 37,908 transcripts annotated to the NR database, 46.22% of the sequences were aligned to Pyrus x and 45.40% to Malus domestica, whereas loquat itself had a best match percentage of 0.35%. These results may be due to the lack of transcript data related to loquat in the current NR database, reflecting the urgent need to improve the genetic database for this genus. The rational classification of protein coding is critical to maximize the use of transcripts for functional research. The results of the COG analysis showed that the R function (general function prediction only) constituted the greatest proportion, followed by the K function (transcription), L function (replication, recombination and repair) and T function (signal transduction mechanisms),which was similar to the results reported by Gong [17]. This result indicated that the gene expression of loquat under low-temperature stress is related to the above functions and suggested that the use of transcriptome sequencing technology is an effective method for the study of functional genes.

The results of this study provide a new reference for loquat transcription. However, analysis of the loquat transcriptome was not comprehensive, and gene expression and metabolic pathways associated with the mechanism underlying the cold stress response of loquat require further analysis.

## Conclusion

This is the first study to perform SMRT sequencing of the FL transcriptome of embryos of young loquat fruit of plants under low-temperature stress. A total of 38,435 transcripts were obtained, 407 lncRNAs were predicted, 24,832 SSRs and 27,905 coding sequences were identified, and 37,993 transcripts were annotated for subsequent analysis. The number and average length of the transcripts were much better than those of previous studies in the loquat transcriptome using only the second-generation sequencing technique. SMRT sequencing is a useful and effective tool for acquiring reliable full-length transcripts of loquat. This work will facilitate research on the functional identification of genes and elucidation of the molecular mechanism underlying the cold stress response in loquat.
